# Supplementary material for: Phenolic Acids and Flavonoids Play Important Roles in Flower Bud Differentiation in Mikania micrantha: Transcriptomics and Metabolomics
Source: Int J Mol Sci. 2023 Nov 21;24(23):16550. doi: 10.3390/ijms242316550 (PMC10705899; doi:10.3390/ijms242316550)
Supplement: Supplementary file 1 [file ijms-24-16550-s001.zip › Supplementary Figure S1.pdf]

*Supplementary Material*

# Phenolic Acids and Flavonoids Play Important Roles in Flower Bud Differentiation in *Mikania micrantha*: Transcriptomics and Metabolomics

**Ling Pei [1], Yanzhu Gao [1], Lichen Feng [1], Zihan Zhang [1], Naiyong Liu [2], Bin Yang [2,*] and Ning Zhao [1,2,*]**

## 1 Supplementary Figure

The Pearson correlation coefficient between DEGs and DEMs in two different groups is greater than 0.8, and a nine quadrant diagram is drawn.

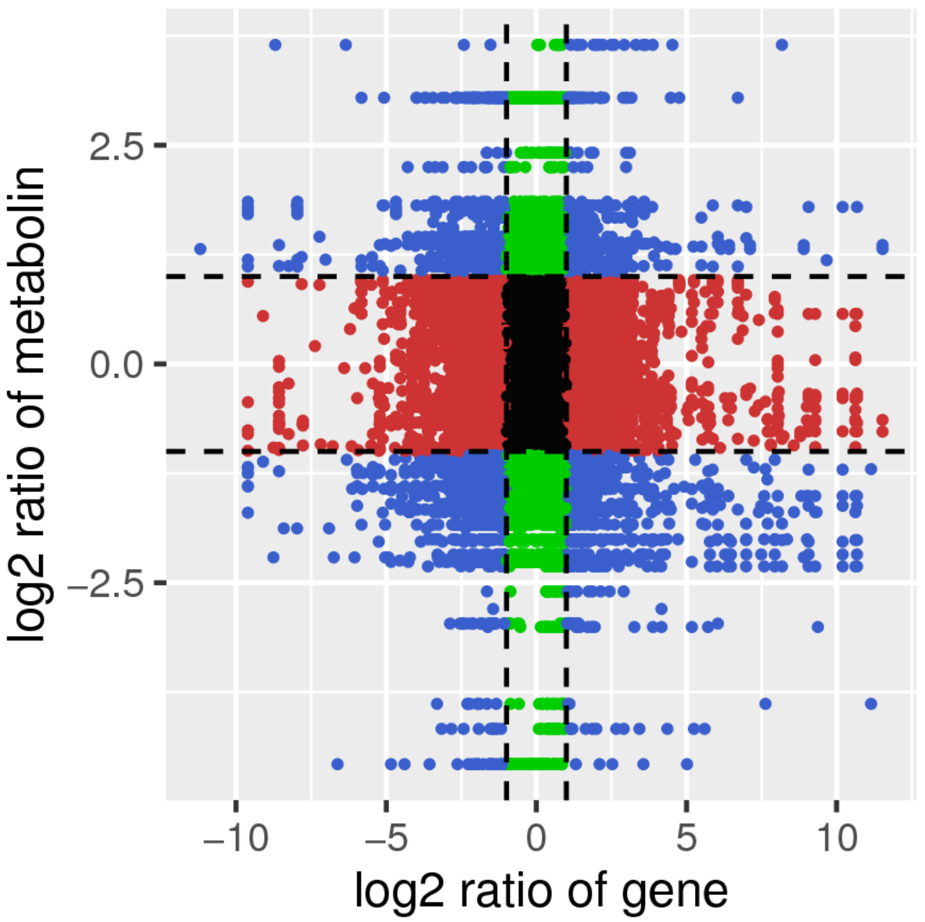

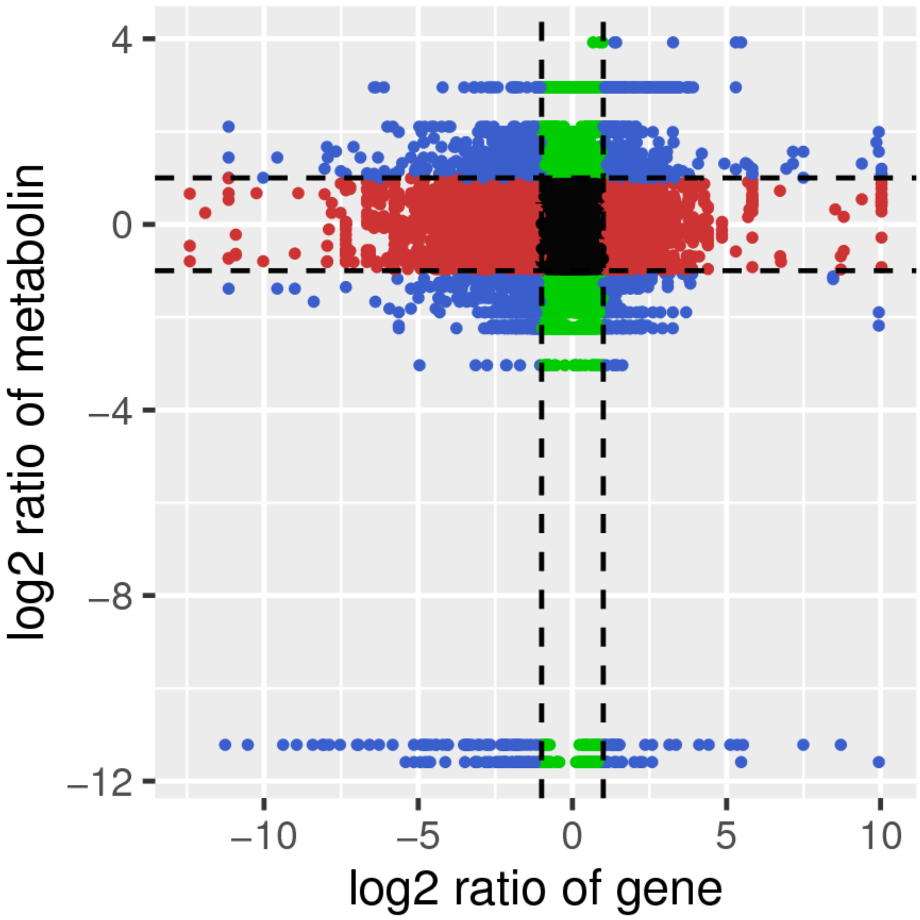


## 2 Supplementary Tables

Supplementary Table S1 | The raw data for the detected metabolic species in our manuscript.

Supplementary Table S2 | Flavonoids metabolites at three altitudes.

Supplementary Table S3 | Phenolic acid metabolites at three altitudes.

Supplementary Table S4 | Transcriptome sequencing data and quality.

Supplementary Table S5 | The KEGG pathway mapped by differentially expressed genes and metabolites at 200 meters vs. 900 meters.

Supplementary Table S6 | The KEGG pathway mapped by differentially expressed genes and metabolites at 1300 meters vs. 900 meters.

Due to formatting constraints, Tables S1-6 can be found in appendix 2.
